# Impact of inflammatory bowel disease on Japanese patients’ quality of life: results of a patient questionnaire survey

**DOI:** 10.1007/s00535-016-1241-x

**Published:** 2016-07-28

**Authors:** Fumiaki Ueno, Yasuo Nakayama, Eiji Hagiwara, Sarina Kurimoto, Toshifumi Hibi

**Affiliations:** 1 Center for Gastroenterology and Inflammatory Bowel Disease, Ofuna Chuo Hospital, Ofuna 6-2-24, Kamakura, Kanagawa Japan; 2 IBD Network, C/O IBD Kaikan, Ringokouen HOUSE #308, Hiragishi 3-jo 5-7-20, Toyohira-ku, Sapporo, 062-0933 Japan; 3 AbbVie GK, Mita 3-5-27, Minato-ku, Tokyo, 108-6302 Japan; 4 Center for Advanced IBD Research and Treatment, Kitasato Institute Hospital, Kitasato University, Shirokane 5-9-1, Minato-ku, Tokyo, 108-8642 Japan

**Keywords:** Inflammatory bowel disease, Quality of life, Ulcerative colitis, Crohn’s disease, Patient organization

## Abstract

**Background:**

Inflammatory bowel disease (IBD) has a significant negative impact on quality of life (QOL); however, the direct impact of IBD on several aspects of patients’ lives is unknown. The IMPACT survey was conducted in Europe in 2010–2011 to determine this impact. We conducted the IMPACT survey in Japan and compared the results between subgroups of patients with ulcerative colitis (UC) and Crohn’s disease (CD).

**Methods:**

The 52-item IMPACT survey questionnaire assessing treatment and the impact of IBD on patients’ lives was translated into Japanese and administered to IBD patients recruited through patient advocacy groups.

**Results:**

Between June 2013 and January 2014, 172 Japanese IBD patients completed the questionnaire (including 84 UC and 83 CD patients). Half of all patients (84/172, 48.8 %) were satisfied with their treatment plan, and half of those who had undergone surgery were satisfied with the outcome (46/87, 52.9 %). Although 34.9 % (60/172) of patients had not been hospitalized in 5 years, 50.0 % (86/172) had been hospitalized for more than 10 days. During the most recent flare, 49.4 % (85/172) of patients had to reschedule appointments because of IBD. Moreover, 32.0 % (55/172) of patients had to make adjustments such as working part-time or at home to avoid taking sick days; 35.5 % (61/172) of patients felt that they had lost a job because of IBD.

**Conclusions:**

Our survey results indicate that IBD patients’ lives and social activities are affected by the deterioration of QOL due to IBD and its symptoms.

**Electronic supplementary material:**

The online version of this article (doi:10.1007/s00535-016-1241-x) contains supplementary material, which is available to authorized users.

## Introduction

Inflammatory bowel disease (IBD) is a group of chronic and recurring inflammatory conditions of the intestine of unknown cause and primarily refers to ulcerative colitis (UC) and Crohn’s disease (CD). As of December 2014 in Japan, 170,781 patients with UC and 40,885 patients with CD were receiving treatment, and the number of patients with IBD is increasing [1]. Because these diseases are intractable and require long-term therapy, patients undergo not only physical strain but also deteriorated quality of life (QOL), both mentally and socially [2–5].

As the need for an appropriate QOL assessment for patients with IBD is increasing, a questionnaire survey of IBD patients, the IMPACT survey was conducted between 2010 and 2011 in 25 European countries under the initiative of the European Federation of Crohn’s and Ulcerative Colitis Associations (EFCCA) [6, 7]. The European IMPACT survey studied IBD treatment and the impact of IBD on patients’ lives and social activities based on survey responses from 4670 patients with IBD.

Similar to the European IMPACT survey, we conducted a survey in Japan; the results are reported here. The aim of this survey was to investigate IBD treatment in Japan and the impact of IBD on patients’ lives and social activities. Also, we compared the results between the subgroups of patients with UC and patients with CD.

## Methods

### IMPACT survey

The questionnaire is a Japanese translation of the English version originally developed by the EFCCA and used in the European IMPACT survey (http://www.efcca.org/index.php/our-activities/ended-projects/51-join-the-fight-against-ibd-project). The English version of the European IMPACT survey questionnaire was developed by the EFCCA, with the support of AbbVie Inc. (North Chicago, IL, USA), and was reviewed by health informaticians. The Japanese translation was examined by one of the authors (F.U.) before its distribution to patients by mail and online.

The questionnaire consists of 52 questions divided into six categories: (1) your experience with IBD, (2) healthcare, (3) the impact that IBD has on your life, (4) overall work impact, (5) overall life impact, and (6) the role of patient organizations. Many of the questions were sourced from validated, published, and peer-reviewed academic surveys or from national IBD association surveys conducted in the past.

### Subjects and methods

The questionnaire survey was conducted via mail or online with the support of the IBD Network, a patient association in Japan, during the period from 1 June 2013 to 31 January 2014. The answers were anonymous and the questionnaires were handled in such a way that personal information was not linked to patient data. Participants were required to be IBD patients, but were otherwise not excluded by any characteristics, such as age or sex. In most questions, participants were allowed to tick one applicable option.

The online survey recruited patients with IBD nationwide who belong to the IBD Network. The IBD Network posted the information on its website and distributed leaflets to its patient members to disseminate the survey information. To avoid duplication as much as possible, each participant was required to register his or her e-mail address; a website link was sent to guide the participant to the questionnaire form. The survey data were managed by the EFCCA. The paper survey was sent to 100 patients who belong to the IBD Network. Survey collection and aggregation were performed by Sunplanet Co., Ltd. (Tokyo, Japan). The surveys collected by Sunplanet were translated into English and sent to the EFCCA.

Data analysis was outsourced from the EFCCA to a research consultant, Survey Solutions Ltd. (Teddington, Middlesex, UK). The analysis results sent to the EFCCA were forwarded to us for interpretation. Each participant gave his or her permission to use the survey data for research and disclose the results after being notified of their complete anonymity.

## Results

### Respondents

Responses were obtained from 172 patients with IBD. Ninety-eight online and 74 paper surveys were collected; the retrieval rate was 74 % for the paper surveys. The respondents consisted of 84 patients with UC, 83 patients with Crohn’s disease, one patient with unclassifiable disease and four patients who did not answer this question (Table 1, Q1). In all, 114 participants were male and 55 were female; three participants did not answer this question. The majority of respondents (94 patients) were in the 35- to 54-year-old age group. Regarding disease activity, 79 patients (45.9 %) were in remission, 55 patients (32.0 %) were having periodic flare-ups and 20 patients (11.6 %) had chronically active disease (Table 1, Q16).

**Table 1:** Respondent characteristics (*N* = 172)

	No.	%
Sex		
Male	114	66.3
Female	55	32.0
No answer	3	1.7
Age group, years		
12–15	2	1.7
16–18	2	1.7
19–24	5	2.9
25–34	29	16.9
35–44	47	27.3
45–54	47	27.3
55–64	31	18.0
65+	7	4.1
No answer	2	1.7
Q1 type of IBD		
Crohn’s disease	83	48.3
Ulcerative colitis	84	48.8
Unclassified	1	0.6
No answer	4	2.3
Q16 disease activity		
In remission/not flaring	79	45.9
Chronically active	20	11.6
Active periodic flare-up	55	32.0
Not applicable/don’t know	3	1.7

*IBD* inflammatory bowel disease

### Patient experience with IBD

**Fig. 1:**
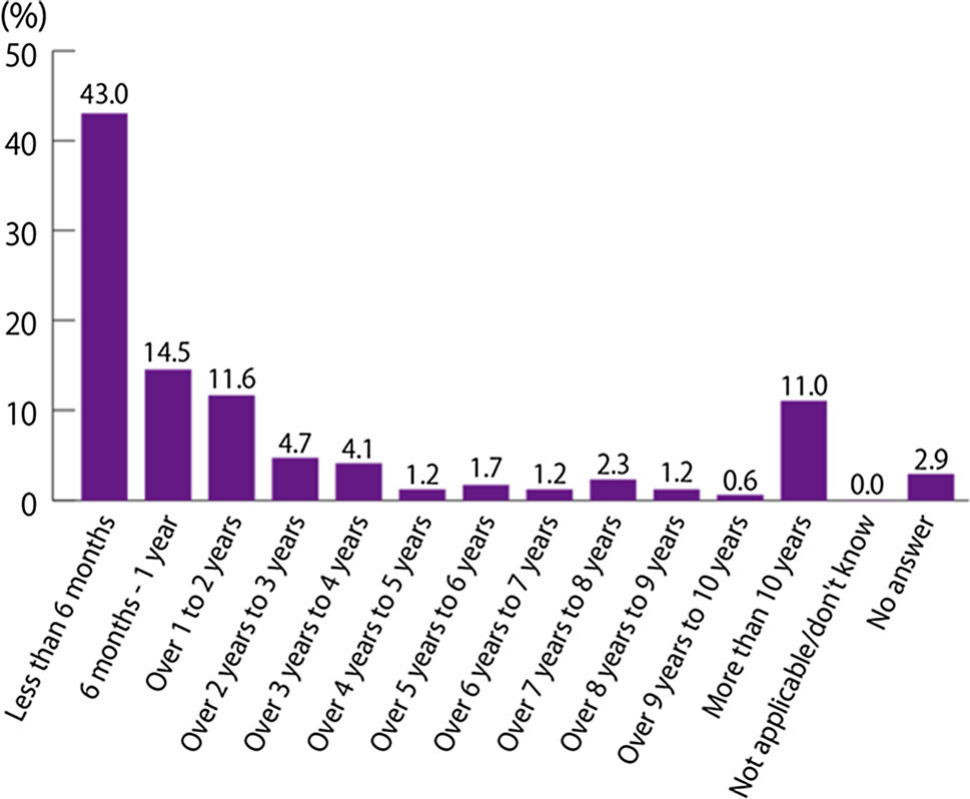
How long after you first began to notice symptoms (that you now recognise as related to IBD) did it take to receive your final diagnosis? (Q2, *N* = 172).* IBD* inflammatory bowel disease

**Fig. 2:**
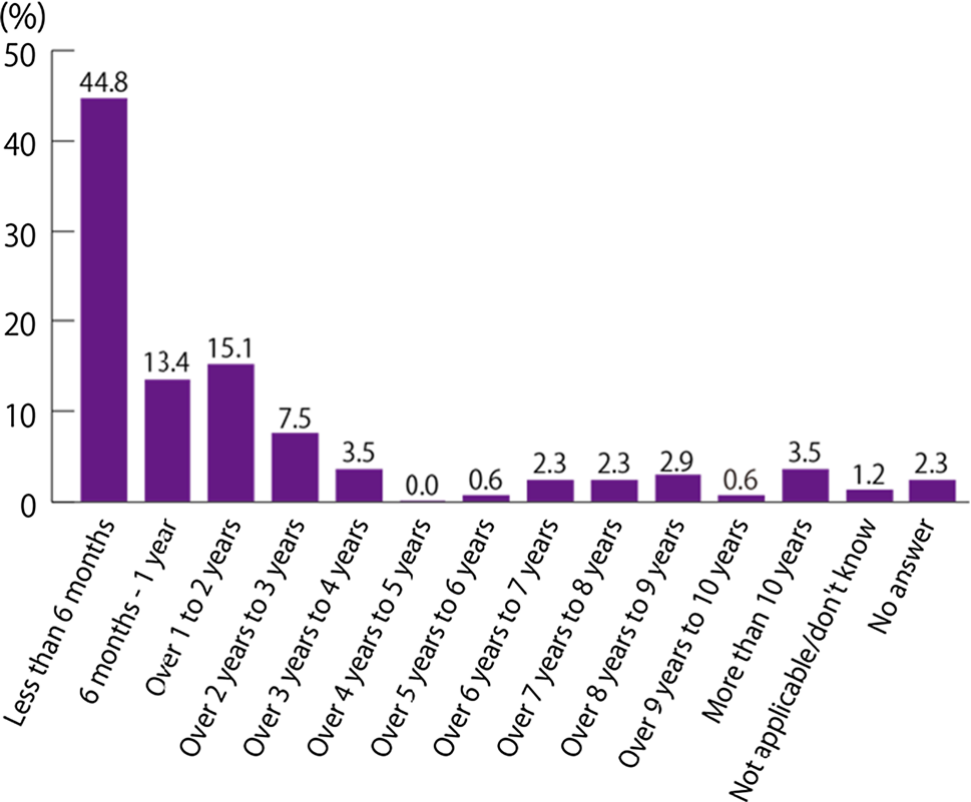
How long after your IBD-related symptoms began did it take for you to first see a specialist (e.g. gastroenterologist, nurse, IBD specialist) who was familiar with IBD? (Q3, *N* = 172).* IBD* inflammatory bowel disease

The time between noticing the first symptoms and receiving a final diagnosis was less than 6 months for 74 patients (43.0 %), representing the most common response. A final diagnosis was made within 1 year for 99 patients (57.6 %). Conversely, it took 5 years or longer to arrive at a final diagnosis for 31 patients (18.0 %) (Fig. 1, Q2). The time between noticing IBD-related symptoms and seeing an IBD specialist was less than 6 months for 77 patients (44.8 %), representing the most common response. The majority of patients (100 patients; 58.1 %) saw a specialist within 1 year (Fig. 2, Q3).

### Healthcare

Among the respondents, 82 patients (47.7 %) did not have a history of surgery for IBD, while 24 patients (14.0 %) had undergone one surgery and 63 patients (36.6 %) had underwent two or more surgeries (Table S1, Q6). Regarding patient satisfaction with operative outcome, 46 patients were very or somewhat satisfied (Table 2, Q7), accounting for 52.9 % of the 87 patients who underwent surgery at least once. Regarding hospitalisation in the past 5 years, 60 patients (34.9 %) were not hospitalised at all, 36 patients (20.9 %) spent 1–30 days in the hospital, 44 patients (25.6 %) spent 31–100 days in the hospital and 20 patients (11.6 %) had been hospitalised for more than 100 days (Table S1, Q8).

**Table 2:** Satisfaction with treatment and communication with healthcare professionals (*N* = 172)

	No.	%	
Q7 How satisfied are you with the outcomes of your operation(s)?			
Very satisfied	18	10.5	
Somewhat satisfied	28	16.3	
Neither satisfied nor dissatisfied	25	14.5	
Somewhat dissatisfied	11	6.4	
Very dissatisfied	4	2.3	
Not applicable/don’t know	69	40.1	
No answer	17	9.9	
Q10 Overall, how satisfied are you with your current treatment plan for IBD?			
Very satisfied	24	14.0	
Somewhat satisfied	60	34.9	
Neither satisfied nor dissatisfied	55	32.0	
Somewhat dissatisfied	16	9.3	
Very dissatisfied	5	2.9	
Not applicable/don’t know	0	0.0	
No answer	12	7.0	
Q12 How often, after an appointment with a gastroenterologist, do you wish that he or she should have asked you more probing questions to better understand your disease status?			
Always (100 % of the time)	9	5.2	
Most of the time (75–99 %)	15	8.7	
Much of the time (50–74 %)	18	10.5	
Sometimes (25–49 %)	76	44.2	
Hardly ever/never (less than 25 % of the time)	46	26.7	
Not applicable/don’t know	5	2.9	
No answer	3	1.7	
Q13 How often, after an appointment with a gastroenterologist, do you feel you did not get to tell the doctor something about your IBD that may have been important?			
Always (100 % of the time)	4	2.3	
Most of the time (75–99 %)	11	6.4	
Much of the time (50–74 %)	16	9.3	
Sometimes (25–49 %)	48	27.9	
Hardly ever/never (less than 25 % of the time)	75	43.6	
Not applicable/don’t know	11	6.4	
No answer	7	4.1	
Q14 Do you believe that you have adequate access to your IBD professional? For example, can you get an appointment to see your IBD healthcare professional on a timely basis?			
Yes	136	79.1	
No	20	11.6	
Don’t know	14	8.1	
No answer	2	1.2	

Q15d Which professional(s) do you feel best understands the full picture of how IBD impacts your life—not just the medical problem? (Tick all that apply)			
	Applicable, No. (%)	Not applicable, No. (%)	No answer, No. (%)
Specialists/gastroenterologist	107 (62.2)	62 (36.0)	3 (1.7)
General physician/clinic	32 (18.6)	137 (79.7)	3 (1.7)
Nurse	34 (19.8)	135 (78.5)	3 (1.7)
Counsellor or psychologist	5 (2.9)	164 (95.3)	3 (1.7)
Other	7 (4.1)	162 (94.2)	3 (1.7)
Don’t know/not applicable	36 (20.9)	133 (77.3)	3 (1.7)

*IBD* inflammatory bowel disease

**Fig. 3:**
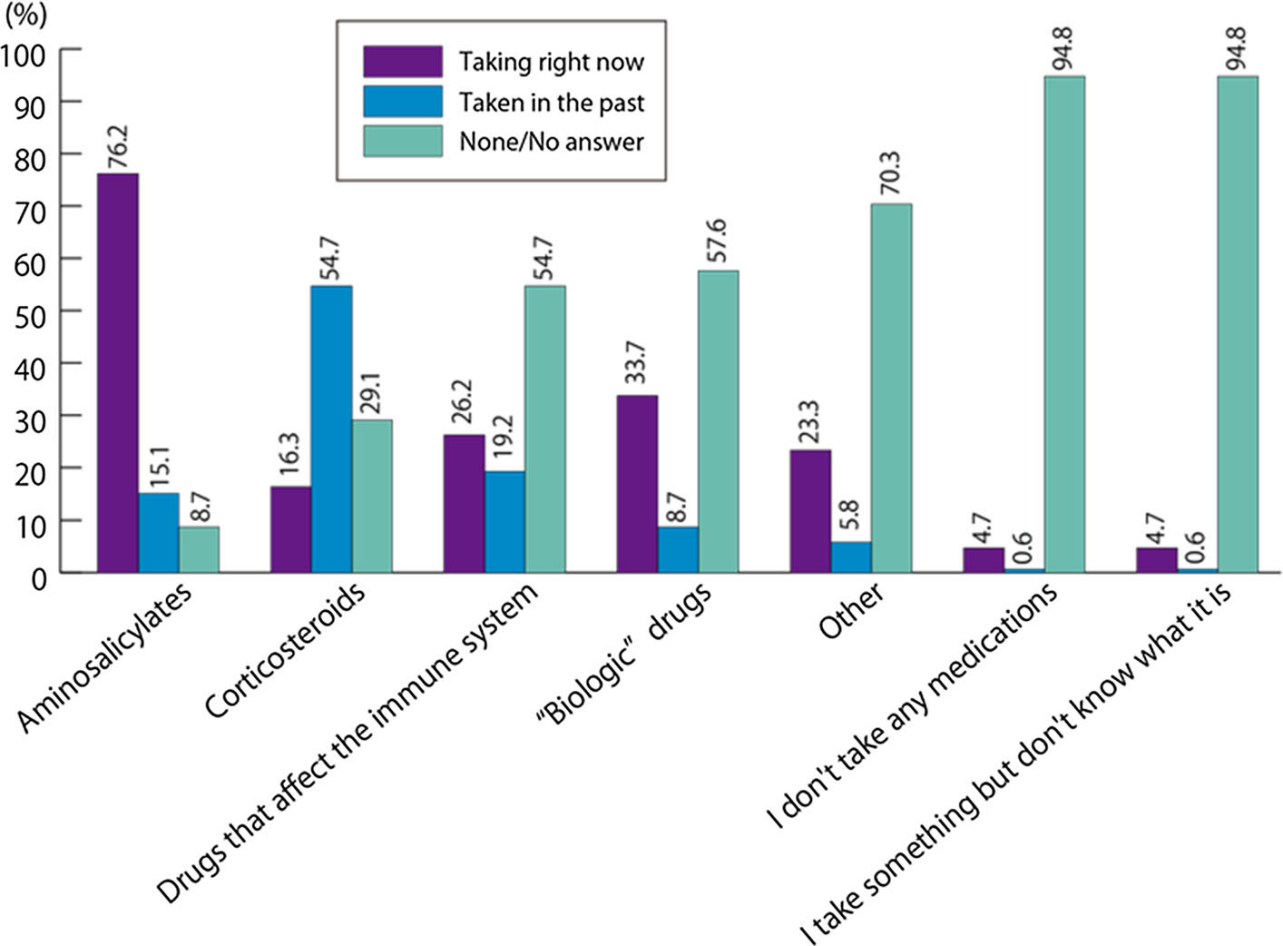
Which of the following types of medicine are you taking right now? Or, have you taken in the past? (Q9, *N* = 172)

Aminosalicylates were the most commonly used medications; 131 patients (76.2 %) were currently receiving aminosalicylates at the time of the survey and 26 patients (15.1 %) had received aminosalicylates in the past. Regarding other concomitant medications, 28 patients (16.3 %) were currently being treated with corticosteroids at the time of the survey and 94 patients (54.7 %) had been treated with corticosteroids in the past; 45 patients (26.2 %) were being treated with immunomodulators at the time of the survey and 33 patients (19.2 %) had been treated with immunomodulators in the past; 58 patients (33.7 %) were currently being treated with biologic drugs and 15 patients (8.7 %) had been treated with biologic drugs in the past. There were eight patients (4.7 %) who were not taking any medication at the time of the survey (Fig. 3, Q9).

Approximately half of the respondents were satisfied with their current IBD treatment plan; 24 patients (14.0 %) were very satisfied and 60 patients (34.9 %) were somewhat satisfied (Table 2, Q10). There were 147 patients (85.5 %) who reported that they had access to a gastroenterology specialist at the clinic where they were being treated (Q11, data not shown).

After an appointment with a gastroenterologist, 118 patients (68.6 %) sometimes (or more often) thought that the physician should have asked more probing questions to better understand their disease status (Table 2, Q12). In addition, 79 patients (45.9 %) sometimes (or more often) felt that they did not get to tell the physician something potentially important about their IBD status (Table 2, Q13).

Among the respondents, 136 patients (79.1 %) felt that access to their IBD professional was adequate (Table 2, Q14). In response to questions about communication with medical professionals, 114 patients (66.3 %) answered that the best range of options for communication was available with an IBD specialist/gastroenterologist, 38 patients (22.1 %) selected a nurse, 31 patients (18.0 %) selected a general physician/clinic and one patient (0.6 %) selected a counsellor (Q15a, data not shown). With regard to medical professionals who best understand not just the medical problems but also the entire picture of how IBD impacts their life, 107 patients (62.2 %) chose an IBD specialist/gastroenterologist, 34 patients (19.8 %) chose a nurse, 32 patients (18.6 %) chose a general physician/clinic and five patients (2.9 %) chose a counsellor (Table 2, Q15d).

### Impact that IBD has on your life

During the current or most recent flare, 85 patients (49.4 %) sometimes or more frequently had to cancel or reschedule an engagement or meeting because of IBD (Table 3, Q19). During the current or most recent period not experiencing a flare, 90 patients (52.3 %) hardly ever/never had to cancel or reschedule an engagement or meeting because of IBD, and 50 patients (29.1 %) had to sometimes or more frequently cancel or reschedule an engagement or meeting because of IBD (Table S2, Q27).

**Table 3:** Impact of IBD on work and life (*N* = 172)

	No.	%	
Q18 How many flare-ups would you say you have experienced in the last 2 years?			
0 (None)	56	32.6	
1	33	19.2	
2	25	14.5	
3	17	9.9	
4	5	2.9	
5	5	2.9	
6	1	0.6	
7	0	0.0	
8	0	0.0	
9	0	0.0	
10	1	0.6	
More than 10	4	2.3	
My condition is always (chronically) actively flaring	20	11.6	
No answer	5	2.9	
Q19 During your current or most recent flare, how often do/did you have to cancel or reschedule an engagement, meeting, etc. because of your IBD? (Select the option that most closely represents your own experience)			
Always (100 % of the time)	12	7.0	
Most of the time (75–99 %)	12	7.0	
Much of the time (50–74 %)	24	14.0	
Sometimes (25–49 %)	37	21.5	
Hardly ever/never (less than 25 % of the time)	56	32.6	
Not applicable/don’t know	27	15.7	
No answer	4	2.3	
Q36 Please indicate any adjustments you have made to your working life, purely to avoid the need to take sick days as a result of your IBD:			
I have not made any such adjustments	102	59.3	
Working flexible hours	14	8.1	
Working from home	17	9.9	
Working part-time	24	14.0	
No answer	15	8.7	

	Applicable, No. (%)	Not applicable, No. (%)	No answer, No. (%)
Q38 If you have been absent from work due to your IBD, what was this primarily due to? (Tick all that apply)			
Hospital/emergency department visit	39 (22.7)	116 (67.4)	17 (9.9)
Doctor appointment	87 (50.6)	68 (39.5)	17 (9.9)
Incontinence or fear of incontinence	13 (7.6)	142 (82.6)	17 (9.9)
Cramping or painful abdomen	40 (23.3)	115 (66.9)	17 (9.9)
Fear of toilet frequency interfering with work activities	28 (16.3)	127 (73.8)	17 (9.9)
Fear of toilet frequency bringing attention to my condition from colleagues	6 (3.5)	149 (86.6)	17 (9.9)
Fatigue and/or not enough energy to get through the day	54 (31.4)	101 (58.7)	17 (9.9)
Worry about gas pressure, discomfort	20 (11.6)	135 (78.5)	17 (9.9)
Worry/fear of potential for embarrassment	7 (4.1)	148 (86.0)	17 (9.9)
Rectal/anal pain or burning	8 (4.6)	147 (85.5)	17 (9.9)
Volume of blood in bleeding episode	13 (7.6)	142 (82.6)	17 (9.9)
I have never been absent from work due to IBD	8 (4.6)	147 (85.5)	17 (9.9)
Not applicable/other	31 (18.0)	124 (72.1)	17 (9.9)

	No.	%	
Q39 Have you received or heard of complaints or unfair comments from your superiors and/or colleagues about your performance at work in relation to your illness?			
No	112	65.1	
Yes	46	26.7	
No answer	14	8.1	
Q40 Do you believe you have been discriminated in the workplace as a direct result of your IBD?			
No	120	69.8	
Yes	38	22.1	
No answer	14	8.1	

	Applicable, No. (%)	Not applicable, No. (%)	No answer, No. (%)
Q41 How does IBD affect your behaviour at work? (Tick all that apply)			
I am quiet or quieter during meetings	24 (13.5)	135 (78.5)	13 (7.6)
I cancel my attendance at meetings at the last minute	12 (7.0)	147 (85.5)	13 (7.6)
I do not participate in work social activities	51 (29.7)	108 (62.8)	13 (7.6)
I am irritable at work	19 (11.0)	140 (81.4)	13 (7.6)
I am less motivated in my work	38 (22.1)	121 (70.3)	13 (7.6)
My IBD does not affect my behaviour at work	46 (26.7)	113 (65.7)	13 (7.6)
Not applicable/other	42 (24.4)	117 (68.0)	13 (7.6)

	No.	%	
Q42 How much do you agree with the following statements? I believe that IBD has negatively affected my career path, opportunities for advancement, income and/or earning potential			
Strongly agree	44	25.6	
Agree	35	20.3	
Neither agree nor disagree	39	22.7	
Disagree	8	4.7	
Strongly disagree	7	4.1	
Not applicable	27	15.7	
No answer	12	7.0	
Q43 I have lost a job (or had to quit a job) because of my IBD			
Strongly agree	39	22.7	
Agree	22	12.8	
Neither agree nor disagree	27	15.7	
Disagree	7	4.1	
Strongly disagree	7	4.1	
Not applicable	58	33.7	
No answer	12	7.0	
Q47 My IBD has negatively affected my ability to perform to my full potential in an educational setting			
Strongly agree	13	7.6	
Agree	40	23.3	
Neither agree nor disagree	49	28.5	
Disagree	17	9.9	
Strongly disagree	15	8.7	
Not applicable	31	18.0	
No answer	7	4.1	

*IBD* inflammatory bowel disease

During the current or most recent flare, 84 patients (48.8 %) did not experience bleeding from the gastrointestinal tract and 84 patients (48.8 %) experienced bleeding at least 1 day a week. Among those who experienced bleeding, 42 patients (24.4 %) had bleeding every day (Table S2, Q20). During the current or most recent period not experiencing a flare, 136 patients (79.1 %) did not experience bleeding from the gastrointestinal tract and 31 patients (18.0 %) experienced bleeding at least 1 day a week. Among those who experienced bleeding, seven patients (4.1 %) had bleeding every day (Table S2, Q28).

During the current or most recent flare, 51 patients (29.7 %) did not experience cramping pain in the abdomen and 116 patients (67.4 %) experienced abdominal pain at least 1 day a week. Of the patients experiencing abdominal pain, 57 patients (33.1 %) experienced abdominal pain every day (Table S2, Q21). During the current or most recent period not experiencing a flare, 99 patients (57.6 %) did not experience cramping pain in the abdomen and 69 patients (40.1 %) experienced abdominal pain at least 1 day a week. Of the patients experiencing abdominal pain, 12 (7.0 %) experienced abdominal pain every day (Table S2, Q29).

During the current or most recent flare, 40 patients (23.3 %) did not feel tired, weak or worn out and 129 patients (75.0 %) felt tired at least 1 day a week. Of the patients who felt tired, weak or worn out, 59 patients (34.3 %) felt tired every day (Table S2, Q22). During the current or most recent period not experiencing a flare, 69 patients (40.1 %) did not feel tired, weak or worn out and 99 patients (57.6 %) felt tired at least 1 day a week. Of the patients who felt tired, weak or worn out, 18 (10.5 %) felt tired every day (Table S2, Q30).

During the current or most recent flare, 56 patients (32.6 %) did not feel a sudden uncontrollable urge for a bowel movement and 113 patients (65.7 %) had bowel urgency at least 1 day a week. Of those experiencing bowel urgency, 49 patients (28.5 %) had bowel urgency every day (Table S2, Q23). During the current or most recent period not experiencing a flare, 91 patients (52.9 %) did not feel a sudden uncontrollable urge for a bowel movement and 77 patients (44.8 %) had bowel urgency at least 1 day a week. Of the patients experiencing bowel urgency, 14 (8.1 %) had bowel urgency every day (Table S2, Q31).

During the current or most recent flare, 26 patients (15.1 %) did not experience runny stools or an episode of diarrhoea and 128 patients (74.4 %) experienced diarrhoea at least once a day. Of the patients experiencing diarrhoea, 48 patients (27.9 %) had diarrhoea 5–10 times a day, representing the most common answer (Table S2, Q24). During the current or most recent period not experiencing a flare, 62 patients (36.0 %) did not experience runny stools or an episode of diarrhoea and 99 patients (57.6 %) experienced diarrhoea at least once a day. Of the patients experiencing diarrhoea, 44 (25.6 %) had diarrhoea one or two times a day, representing the most common answer (Table S2, Q32).

During the current or most recent flare, 69 patients (40.1 %) sometimes or more frequently had to abruptly stop or leave a conversation, meeting or activity because of symptoms of IBD (Table S2, Q25). During the current or most recent period not experiencing a flare, 36 patients (20.9 %) sometimes or more frequently had to abruptly stop or leave a conversation, meeting or activity because of symptoms of IBD (Table S2, Q33).

Eighty-one patients (47.1 %) felt that their lives were significantly or somewhat negatively affected by symptoms of IBD, even between flares (Table S2, Q26). During a period not experiencing a flare, 126 patients (73.3 %) worried sometimes or more frequently about when the next flare would occur. Of these patients, 35 (20.3 %) worried about the next flare all the time (Table S2, Q34).

### Work impact

Regarding the overall work impact, 124 patients (72.1 %) feel stressed or pressured about taking sick time from work because of IBD (Table S2, Q35). Fifty-five patients (32.0 %) made adjustments in their working life to avoid taking sick days: 14 patients (8.1 %) work flexible hours, 17 patients (9.9 %) work from home and 24 patients (14.0 %) work part-time (Table 3, Q36). There were 111 patients (64.5 %) who had taken at least one sick day from work because of IBD in the past year; 25 patients (14.5 %) had been absent for more than 25 days (Table S2, Q37). The most common reasons for being absent from work were a doctor’s appointment (87 patients; 50.6 %), fatigue and/or not enough energy to get through the day (54 patients; 31.4 %), cramping or painful abdomen (40 patients; 23.3 %) and emergency department visits (39 patients; 22.7 %). Patients were also absent because of worries about symptoms instead of actually having symptoms, such as a fear of toilet frequency interfering with work (28 patients; 16.3 %) and worries about gas pressure or discomfort (20 patients; 11.6 %) (Table 3, Q38).

Forty-six patients (26.7 %) had received or heard complaints or unfair comments from superiors and/or colleagues about their work performance in relation to their illness (Table 3, Q39). Some felt they were discriminated in the workplace as a direct result of IBD (38 patients; 22.1 %) (Table 3, Q40). The most commonly reported impact of IBD on workplace behaviour was nonparticipation in work social activities (51 patients; 29.7 %), followed by less motivated at work (38 patients; 22.1 %), being quiet during meetings (24 patients; 13.5 %) and irritability at work (19 patients; 11.0 %). However, 46 patients (26.7 %) answered that their behaviours at work were unaffected by IBD (Table 3, Q41). As many as 79 patients (45.9 %) felt that IBD negatively affected their career path, opportunities for advancement, income and/or earning potential (Table 3, Q42). Sixty-one patients (35.5 %) felt that they had lost a job or had to quit a job because of IBD (Table 3, Q43).

### Overall life impact

Regarding the impact of IBD on overall life, 44 patients (25.6 %) felt that IBD had prevented them from pursuing an intimate relationship, and 38 patients (22.1 %) felt that IBD had caused an intimate relationship to end. In addition, 40 patients (23.3 %) felt that IBD had prevented them from making and/or keeping friends (Table S3, Q44–46).

Fifty-three patients (30.8 %) reported that IBD had negatively affected their ability to perform to their full potential in an educational setting (Table 3, Q47). In all, 108 patients (62.8 %) frequently considered the availability of toilets when planning to attend an event or meeting, 124 patients (72.1 %) worried about the ready availability of a toilet when going somewhere new, 30 patients (17.4 %) kept a list of clean, accessible toilets and considered it when leaving home, 58 patients (33.7 %) had to be rude to people at times in order to gain access to a toilet to prevent an accident, 10 patients (5.8 %) reported that other people had sometimes joked about them when they urgently needed a toilet and 43 patients (25.0 %) frequently woke from sleeping as a result of pain from IBD (Table S4, Q48).

The first time they met another person with IBD, 61 patients (35.5 %) felt more optimistic, positive and/or hopeful about the future, 14 patients (8.1 %) felt more pessimistic, negative and/or less hopeful about the future and 88 patients (51.2 %) felt neither more optimistic nor more pessimistic (Table S4, Q49).

The majority of the respondents (130 patients; 75.6 %) were engaged in an IBD patient association (Table S4, Q50); 73 patients (42.4 %) attend local or national patient meetings, 108 patients (62.8 %) are members of their national IBD association, 73 patients (42.4 %) receive patient information leaflets from their national IBD association, 13 patients (7.6 %) call a helpline or e-mail their national IBD association, 58 patients (33.7 %) subscribe to newsletters or magazines from their national IBD association, 39 patients (22.7 %) volunteer to help their national IBD association, 13 patients (7.6 %) help their national IBD association with fundraising, 38 patients (22.1 %) became a leader or joined a committee within their national IBD association and two patients (1.2 %) became an EFCCA delegate or worked within an EFCCA project team (Table S4, Q51). Among the respondents, 87 patients (50.6 %) felt that being part of the patient association improved their life in general as someone with IBD (Table S3, Q52).

### Subgroup analysis: ulcerative colitis vs. Crohn’s disease

In a subgroup analysis, we compared the results between respondents with UC (84 patients) and CD (83 patients). Regarding disease activity, 38.1 % (32/84) and 55.4 % (46/83) of respondents, respectively, were in remission, 35.7 % (30/84) and 30.1 % (25/83) were having periodic flare-ups and 11.9 % (10/84) and 12.0 % (10/83) had chronically active disease. Table 4 shows the results of the questions for which the differences in the proportions between the two groups exceeded 20 %.

**Table 4:** Comparison of ulcerative colitis and Crohn’s disease: the items for which the differences between the groups were more than 20 %

	Ulcerative colitis (*N* = 84)		Crohn’s disease (*N* = 83)			
	No.	(%)	No.	(%)		
Q2 How long after you first began to notice symptoms (that you now recognise as related to IBD) did it take to receive your final diagnosis?						
Less than 6 months	47	(56.0)	27	(32.5)		
6 months–1 year	12	(14.3)	12	(14.5)		
Over 1 to 5 years	14	(16.7)	23	(27.7)		
Over 5 to 10 years	2	(2.4)	10	(12.0)		
More than 10 years	8	(9.5)	11	(13.3)		
Not applicable	1	(1.2)	0	(0.0)		
Q3 How long after your IBD-related symptoms began did it take for you to first see a specialist (e.g. gastroenterologist, nurse, IBD specialist) who was familiar with IBD?						
Less than 6 months	49	(58.3)	28	(33.7)		
6 months–1 year	11	(13.1)	12	(14.5)		
Over 1 to 5 years	18	(21.4)	27	(32.5)		
Over 5 to 10 years	3	(3.6)	11	(13.3)		
More than 10 years	2	(2.4)	4	(4.8)		
Not applicable	1	(1.2)	1	(1.2)		
Q5-2 I am concerned about the long-term effects of steroids on my health						
Yes	42	(50.0)	19	(22.9)		
No	41	(48.8)	63	(75.9)		
Not applicable	1	(1.2)	1	(1.2)		
Q6 How many surgical operations have you had for IBD and IBD-related medical problems?						
0	64	(76.2)	17	(20.5)		
1–4	16	(19.0)	51	(61.4)		
5–10	4	(4.8)	14	(16.9)		
More than 10	0	(0.0)	1	(1.2)		
Not applicable	0	(0.0)	0	(0.0)		
Q8 Over the last 5 years, how many days in total have you been hospitalised because of IBD symptoms? (Please type in number of days)						
0 days	39	(46.4)	21	(25.3)		
1–10	6	(7.1)	7	(8.4)		
11–50	18	(21.4)	24	(28.9)		
51–100	8	(9.5)	15	(18.1)		
101–200	6	(7.1)	5	(6.0)		
200+	2	(2.4)	8	(9.6)		
Not applicable	5	(6.0)	3	(3.6)		

Q9 Which of the following types of medicine are you taking right now? Or, have you taken in the past?						
	Taking right now, No. (%)	Taken in the past, No. (%)	None/no answer, No. (%)	Taking right now, No. (%)	Taken in the past, No. (%)	None/no answer, No. (%)
Aminosalicylates	65 (77.4)	13 (15.5)	6 (7.1)	64 (77.1)	13 (15.7)	6 (7.2)
Corticosteroids	17 (20.2)	47 (56.0)	20 (23.8)	10 (12.0)	47 (56.6)	26 (31.3)
Drugs that affect the immune system	18 (21.4)	14 (16.7)	52 (61.9)	27 (32.5)	18 (21.7)	38 (45.8)
“Biologic” drugs	7 (8.3)	5 (6.0)	72 (85.7)	50 (60.2)	10 (12.0)	23 (27.7)
Other	17 (20.2)	4 (4.8)	63 (75.0)	23 (27.7)	6 (7.2)	54 (65.1)
I don’t take any medications	4 (4.8)	0 (0.0)	80 (95.2)	3 (3.6)	1 (1.2)	79 (95.2)
I take something but don’t know what it is	4 (4.8)	1 (1.2)	79 (94.0)	2 (2.4)	0 (0.0)	81 (97.6)

	No.	(%)	N	(%)		
Q17 When was your last flare (before this one if you are currently experiencing one)?						
Less than 6 months ago	27	(32.1)	20	(24.1)		
Over 6–12 months ago	10	(11.9)	8	(9.6)		
More than 12 months ago	24	(28.6)	43	(51.8)		
Not applicable	23	(27.4)	12	(14.5)		
Q20 During your current or most recent flare, how many days in a week do/did you experience bleeding from your gastrointestinal tract? (Select the option that most closely represents your own experience)						
0 day	30	(35.7)	53	(63.9)		
1–3 days	13	(15.5)	19	(22.9)		
4–6 days	6	(7.1)	2	(2.4)		
7 days	35	(41.7)	7	(8.4)		
Not applicable	0	(0.0)	2	(2.4)		
Q22 During your current or most recent flare, how many days in a week do/did you feel tired, weak or worn out? (Select the option that most closely represents your own experience)						
0 day	23	(27.4)	17	(20.5)		
1–3 days	29	(34.5)	16	(19.3)		
4–6 days	12	(14.3)	10	(12.0)		
7 days	20	(23.8)	39	(47.0)		
Not applicable	0	(0.0)	1	(1.2)		
Q32 During your current or most recent period NOT experiencing a flare, how many runny stools or episodes of diarrhoea do/did you experience in the course of a typical day? (Select the option that most closely represents your own experience)						
Did not experience	40	(47.6)	21	(25.3)		
1–2 times a day	24	(28.6)	19	(22.9)		
More than 2 times a day	14	(16.7)	40	(48.2)		
Not applicable	6	(7.1)	3	(3.6)		
Q37 In the past year, how many days have you been absent from work for reasons related to your IBD?						
0 day	29	(34.5)	17	(20.5)		
1 day or more	46	(54.8)	64	(77.1)		
Not applicable	9	(10.7)	2	(2.4)		

*IBD* inflammatory bowel disease

Patients with UC were diagnosed or visited a specialist within a relatively short time after first noticing symptoms, and patients with UC experienced more bleeding from the gastrointestinal tract during a flare than patients with CD. Forty-seven patients (56.0 %) with UC were diagnosed within 6 months of first noticing symptoms, whereas this was the case for only 27 patients (32.5 %) with CD (Table 4, Q2). Forty-nine patients (58.3 %) with UC saw a specialist within 6 months of first noticing symptoms, whereas this was the case for only 28 patients (33.7 %) with CD (Table 4, Q3). During the current or most recent flare, 35 patients (41.7 %) with UC had bleeding every day, as opposed to seven patients (8.4 %) with CD (Table 4, Q20). During the current or most recent period not experiencing a flare, 40 patients (47.6 %) with UC did not experience runny stools or an episode of diarrhoea, as opposed to 21 patients (25.3 %) with CD (Table 4, Q32).

Compared to patients with CD, patients with UC were more concerned about the side effects of steroids, and fewer patients with UC had a history of hospitalization or surgery. Forty-two patients (50.0 %) with UC were concerned about the long-term effects of steroids on their health, as opposed to 19 patients (22.9 %) with CD (Table 4, Q5-2). Regarding surgery, 64 patients (76.2 %) with UC and 17 patients (20.5 %) with CD had never undergone surgery; four patients (4.8 %) with UC had undergone five or more surgeries, as opposed to 15 patients (18.0 %) with CD (Table 4, Q6). Regarding hospitalization in the past 5 years, 39 patients (46.4 %) with UC and 21 (25.3 %) with CD had no hospital stays (Table 4, Q8). Regarding medications, fewer patients with UC were taking biologic drugs at the time of the survey compared to patients with CD (8.3 % [7/84] vs. 60.2 % [50/83]) (Table 4, Q9).

Patients with CD tended to have had a longer time since their last flare at the time of the survey, though they also seemed to feel more tired during the flare and have been absent from work because of IBD more often. Forty-three patients (51.8 %) with CD experienced their last flare more than 1 year ago, compared to 24 patients (28.6 %) with UC (Table 4, Q17). During the current or most recent flare, 39 patients (47.0 %) with CD felt tired every day, as opposed to 20 patients (23.8 %) with UC (Table 4, Q22). Sixty-four patients (77.1 %) with CD had taken at least one sick day from work because of IBD in the past year, as opposed to 46 patients (54.8 %) with UC (Table 4, Q37).

## Discussion

Our survey results indicate that the lives and social activities of patients with IBD are affected by the deterioration of QOL due to IBD and its symptoms. In Japan, several previous studies have also investigated QOL and the impact of IBD on patients’ lives and social activities and similarly demonstrated deterioration in QOL associated with IBD [8–17]. Our present survey is unique in that we used a questionnaire that comprised basically the same set of questions as the European IMPACT survey to allow direct comparison between the Japanese and European survey results. Our survey results will not only help improve medical treatment and care for patient with IBD in Japan but also provide useful information for reviewing medical practice for IBD in each country in Europe.

Our study results were similar to those of the European IMPACT survey for most components, including the time to definitive diagnosis, use of medications and life impact of IBD [6, 7]. Despite differences in race, life environment and medical system, the results of our study and the European IMPACT study likely reflect common trends in IBD patients. As in the European IMPACT study, the Japanese survey points out several issues: (1) communication between medical specialists and patients can be further improved, (2) IBD patients often have to take sick days from work and have psychological fear at work because of their illness, (3) IBD has a significant impact on daily life, including constant concern about toilets and difficulty building intimate relationships, and (4) IBD interferes with learning. Patients with IBD have unmet needs; the goal is to improve medical care and the social support system for patients with IBD in Japan.

While the Japanese results were generally similar to the European survey results [6], there were several differences. First, the proportion of patients who were somewhat or very satisfied with the outcomes of IBD-related surgical operations was lower in Japan (52.9 %) than in Europe (72.9 %). This may be partly attributed to the higher percentage of Japanese patients with surgical complications (20.3 %) compared with European patients (14.6 %). In addition, Japanese patients were less satisfied with their current treatment plan for IBD than European patients (48.8 vs 70.3 %). Because the answers to questions about the adequateness of communication with medical professionals were similar between Japanese and European patients, the lower level of satisfaction in Japanese patients cannot be attributed solely to inadequate communication, indicating the presence of some other issue with the medical treatment itself or the medical environment in Japan.

Although a greater percentage of Japanese patients had not been hospitalised in the past 5 years compared with European patients (34.9 vs 15.4 %), more Japanese patients had been hospitalised for more than 10 days compared with European patients (50.0 vs 2.1 %). In addition, 11.6 % of Japanese patients remained in the hospital for more than 100 days, indicating that Japanese patients, once hospitalised, tend to remain in the hospital longer or return frequently.

Regarding healthcare professionals who are available for communication or who have the greatest influence on IBD patients, fewer Japanese patients named a counsellor or clinical psychologist compared with European patients. This indicates that counsellors and clinical psychologists are not sufficiently positioned in Japanese hospitals or are not adequately involved with patients with IBD. As shown in our survey results, IBD patients suffer a great deal of mental strain from their illness; thus, we will have to reconsider how counsellors and clinical psychologists can be involved in their care.

Finally, slightly more Japanese patients with IBD seem to feel stressed or pressured about taking sick time from work because of IBD compared with European patients (72.1 vs 60.5 %). Because Japanese patients do not seem to have particularly high external pressure, such as unfair treatment, discrimination and career path impediment due to IBD, Japanese patients may be too self-critical compared with European patients. Therefore, mental support will be necessary to promote the social participation of patients with IBD in Japan.

Subgroup analysis comparing UC and CD revealed characteristics of each disease based on the differences between the subgroups. UC is associated with more frequent anaemia, bloody stool, and mucous and bloody stool compared with CD, for which the frequency of bloody stool is less frequent. This is attributed to the characteristics of CD, in which lesions do not easily bleed and the rectum is less frequently affected. Thus, the present study results are consistent with the pathology of these conditions [18]. Patients with UC tend to develop intestinal haemorrhage on a daily basis during flare-ups, and many of them visited a specialist and received a definitive diagnosis at a relatively early phase. As described above, this is due to the pathological characteristics of UC, with patients having bloody stool and mucous and bloody stool more often compared with CD. On the other hand, patients with CD tend to have more adverse influences in their daily lives, such as absence from work, which seems to be due to malaise during flare-ups. However, the duration of a flare-up was not long in many patients. UC affects areas near the intestinal lumen, from the mucosal layer to the submucosal layer, representing shallow inflammation. In contrast, CD causes deeper inflammation, often reaching the muscle layer. UC, with shallower inflammation, is generally acknowledged as a milder enterocolitis than CD, which causes deeper inflammation. Thus, CD has a greater impact on labour productivity compared with UC, from a pathological point of view [19–21].

With regard to the therapy, many patients with UC expressed concern about the long-term use of steroids, while the majority of patients with CD received aggressive therapy, including hospitalization, surgical intervention and treatment with biological agents. This reflects the current UC treatment practice that frequently recommends steroids as part of remission induction therapy according to clinical practice guidelines (Japan, USA and EU) [22–24]. It should be noted that steroids are not suitable for maintenance therapy but are highly effective in remission induction. In the real world, patients with steroid dependency are treated with steroids for an extended period, and the associated adverse reactions are a concern [25, 26].

Although our survey offers a broad perspective, there are several limitations to this study. First, the survey participation was free and therefore it was inherently associated with a bias in respondent selection. Second, while not all respondents were attending patient associations, survey recruitment was conducted only within the reach of information from the IBD Network patient association and the respondents may not include those who were not closely related with that association. Third, the majority of respondents were acquired through the online survey site, and the results may not represent those who do not have access to the Internet. Fourth, the Japanese survey sample size was smaller than that of the European IMPACT survey. However, our survey achieved the minimum target of 100, which was also the target in the European survey [6]. Finally, several questions may have had a recall bias, as patients had to recall situations in the past.

Accessibility to the Internet or the IBD network may vary depending upon a patient’s awareness of treatment options and their environment; therefore, our survey may not necessarily reflect the current status of Japanese patients with IBD. Providing patients with a questionnaire during their medical visits, i.e. administering the survey via a healthcare professional, would better reflect their current situation across a broader demographic.

## Conclusions

Our survey results indicate that lives and social activities of IBD patients in Japan are affected by the deterioration of QOL due to IBD and its symptoms. Information on the unmet needs of Japanese patients with IBD may help to provide sufficient medical and administrative support, improve working and educational environments, and increase the awareness of healthcare providers in order to bridge communication gaps between patients and physicians.

## Electronic supplementary material

Below is the link to the electronic supplementary material.

Supplementary material 1 (DOCX 44 kb)
