# Supplementary material for: Impact of inflammatory bowel disease on Japanese patients’ quality of life: results of a patient questionnaire survey
Source: J Gastroenterol. 2016 Jul 28;52(5):555–67. doi: 10.1007/s00535-016-1241-x (PMC5397430; doi:10.1007/s00535-016-1241-x)
Supplement: Supplementary file 1 — Supplementary material 1 (DOCX 44 kb) [file 535_2016_1241_MOESM1_ESM.docx]

**Table S1. Treatment for IBD (N=172)**

|  | No. | % |
| --- | --- | --- |
| Q6 How many surgical operations have you had for IBD and IBD-related medical problems? | | |
| 0 (None) | 82 | 47.7 |
| 1 | 24 | 14.0 |
| 2 | 21 | 12.2 |
| 3 | 15 | 8.7 |
| 4 | 8 | 4.7 |
| 5 | 9 | 5.2 |
| 6 | 4 | 2.3 |
| 7 | 3 | 1.7 |
| 8 | 2 | 1.2 |
| 9 | 0 | 0.0 |
| 10 | 0 | 0.0 |
| More than 10 | 1 | 0.6 |
| No answer | 3 | 1.7 |
| Q8 Over the last 5 years, how many days in total have you been hospitalised because of IBD symptoms? (please type in number of days) | | |
| 0 days | 60 | 34.9 |
| 1–5 | 6 | 3.9 |
| 6–10 | 8 | 4.7 |
| 11–20 | 5 | 2.9 |
| 21–30 | 17 | 9.9 |
| 31–40 | 7 | 4.1 |
| 41–50 | 14 | 8.1 |
| 51–100 | 23 | 13.4 |
| 101–200 | 11 | 6.4 |
| 201–300 | 7 | 4.1 |
| 300+ | 2 | 1.2 |
| No answer | 12 | 7.0 |

IBD, inflammatory bowel disease.

**Table S2. The impact of IBD on work and life (N=172)**

|  | No. | % | | |
| --- | --- | --- | --- | --- |
| Q17 When was your last flare (before this one if you are currently experiencing one)? | | | | |
| Less than a month ago | 18 | 10.5 | | |
| Over 1 month to 3 months ago | 13 | 7.6 | | |
| Over 3 months to 6 months ago | 17 | 9.9 | | |
| Over 6 months to12 months ago | 18 | 10.5 | | |
| More than 12 months ago | 67 | 39.0 | | |
| Not applicable/don't know | 35 | 20.3 | | |
| No answer | 4 | 2.3 | | |
| Q20 During your current or most recent flare, how many days in a week do/did you experience bleeding from your gastrointestinal tract?  (select the option that most closely represents your own experience) | | | | |
| 0 day | 84 | 48.8 | | |
| 1 day | 16 | 9.3 | | |
| 2 days | 8 | 4.7 | | |
| 3 days | 9 | 5.2 | | |
| 4 days | 3 | 1.7 | | |
| 5 days | 5 | 2.9 | | |
| 6 days | 1 | 0.6 | | |
| 7 days | 42 | 24.4 | | |
| No answer | 4 | 2.3 | | |
| Q21 During your current or most recent flare, how many days in a week do/did you experience cramping pain in your abdomen?  (select the option that most closely represents your own experience) | | | | |
| 0 day | 51 | 29.7 | | |
| 1 day | 15 | 8.7 | | |
| 2 days | 12 | 7.0 | | |
| 3 days | 15 | 8.7 | | |
| 4 days | 6 | 3.5 | | |
| 5 days | 10 | 5.8 | | |
| 6 days | 1 | 0.6 | | |
| 7 days | 57 | 33.1 | | |
| No answer | 5 | 2.9 | | |
| Q22 During your current or most recent flare, how many days in a week do/did you feel tired, weak or worn out?  (select the option that most closely represents your own experience) | | | | |
| 0 day | 40 | 23.3 | | |
| 1 day | 14 | 8.1 | | |
| 2 days | 18 | 10.5 | | |
| 3 days | 16 | 9.3 | | |
| 4 days | 8 | 4.7 | | |
| 5 days | 10 | 5.8 | | |
| 6 days | 4 | 2.3 | | |
| 7 days | 59 | 34.3 | | |
| No answer | 3 | 1.7 | | |
| Q23 During your current or most recent flare, how many days in a week do/did you feel a sudden, uncontrollable urge for a bowel movement?  (select the option that most closely represents your own experience) | | | | |
| 0 day | 56 | 32.6 | | |
| 1 day | 15 | 8.7 | | |
| 2 days | 10 | 5.8 | | |
| 3 days | 21 | 12.2 | | |
| 4 days | 7 | 4.1 | | |
| 5 days | 5 | 2.9 | | |
| 6 days | 6 | 3.5 | | |
| 7 days | 49 | 28.5 | | |
| No answer | 3 | 1.7 | | |
| Q24 During your current or most recent flare, how many runny stools or episodes of diarrhoea do/did you experience in the course of a typical day?  (select the option that most closely represents your own experience) | | | | |
| Did not experience | 26 | 15.1 | | |
| 1-2 times a day | 16 | 9.3 | | |
| 3-4 times a day | 29 | 16.9 | | |
| 5-10 times a day | 48 | 27.9 | | |
| 11-15 times a day | 22 | 12.8 | | |
| More than 15 times a day | 13 | 7.6 | | |
| Not applicable/don’t know | 14 | 8.1 | | |
| No answer | 4 | 2.3 | | |
| Q25 During your current or most recent flare, do/did you find that you frequently had to abruptly stop or leave a conversation, meeting or activity to deal with symptoms of your bowel disease? (tick the box that most closely represents your own experience) | | | | |
| No | 99 | 57.6 | | |
| Yes | 69 | 40.1 | | |
| No answer | 4 | 2.3 | | |
| Q26 In between flares, my life is still significantly negatively impacted by symptoms of IBD compared to people without IBD | | | | |
| Very unaffected – when I’m in remission, I never think of my IBD | 19 | | 11.0 | |
| Slightly unaffected – with the exception of a few activities which I avoid, I get on with my life | 68 | | 39.5 | |
| Somewhat affected – I avoid doing a lot of things in case I get a flare up | 37 | | 21.5 | |
| Significantly affected – it is always on my mind | 44 | | 25.6 | |
| No answer | 4 | | 2.3 | |
| Q27 During your current or most recent period NOT experiencing a flare, how often do/did you have to cancel or reschedule an engagement, meeting, etc. because of your IBD?  (select the option that most closely represents your own experience) | | | | |
| Always (100% of the time) | 5 | 2.9 | | |
| Most of the time （75%–99%） | 4 | 2.3 | | |
| Much of the time (50%–74%) | 10 | 5.8 | | |
| Sometimes (25%–49%) | 31 | 18.0 | | |
| Hardly ever/never (less than 25% of the time) | 90 | 52.3 | | |
| Not applicable/don’t know | 28 | 16.3 | | |
| No answer | 4 | 2.3 | | |
| Q28 During your current or most recent period NOT experiencing a flare, how many days in a week do/did you experience bleeding from your gastrointestinal tract?  (select the option that most closely represents your own experience) | | | | |
| 0 day | 136 | 79.1 | | |
| 1 day | 15 | 8.7 | | |
| 2 days | 3 | 1.7 | | |
| 3 days | 3 | 1.7 | | |
| 4 days | 1 | 0.6 | | |
| 5 days | 2 | 1.2 | | |
| 6 days | 0 | 0.0 | | |
| 7 days | 7 | 4.1 | | |
| No answer | 5 | 2.9 | | |
| Q29 During your current or most recent period NOT experiencing a flare, how many days in a week do/did you experience cramping pain in your abdomen?  (select the option that most closely represents your own experience) | | | | |
| 0 day | 99 | 57.6 | | |
| 1 day | 24 | 14.0 | | |
| 2 days | 17 | 9.9 | | |
| 3 days | 8 | 4.7 | | |
| 4 days | 7 | 4.1 | | |
| 5 days | 1 | 0.6 | | |
| 6 days | 0 | 0.0 | | |
| 7 days | 12 | 7.0 | | |
| No answer | 4 | 2.3 | | |
| Q30 During your current or most recent period NOT experiencing a flare, how many days in a week do/did you feel tired, weak or worn out?  (select the option that most closely represents your own experience) | | | | |
| 0 day | 69 | 40.1 | | |
| 1 day | 22 | 12.8 | | |
| 2 days | 29 | 16.9 | | |
| 3 days | 18 | 10.5 | | |
| 4 days | 5 | 2.9 | | |
| 5 days | 5 | 2.9 | | |
| 6 days | 2 | 1.2 | | |
| 7 days | 18 | 10.5 | | |
| No answer | 4 | 2.3 | | |
| Q31 During your current or most recent period NOT experiencing a flare, how many days in a week do/did you feel a sudden, uncontrollable urge for a bowel movement?  (select the option that most closely represents your own experience) | | | | |
| 0 day | 91 | 52.9 | | |
| 1 day | 37 | 21.5 | | |
| 2 days | 12 | 7.0 | | |
| 3 days | 10 | 5.8 | | |
| 4 days | 3 | 1.7 | | |
| 5 days | 1 | 0.6 | | |
| 6 days | 0 | 0.0 | | |
| 7 days | 14 | 8.1 | | |
| No answer | 4 | 2.3 | | |
| Q32 During your current or most recent period NOT experiencing a flare, how many runny stools or episodes of diarrhoea do/did you experience in the course of a typical day?  (select the option that most closely represents your own experience) | | | | |
| Did not experience | 62 | 36.0 | | |
| 1-2 times a day | 44 | 25.6 | | |
| 3-4 times a day | 22 | 12.8 | | |
| 5-10 times a day | 25 | 14.5 | | |
| 11-15 times a day | 5 | 2.9 | | |
| More than 15 times a day | 3 | 1.7 | | |
| Not applicable/don’t know | 8 | 4.7 | | |
| No answer | 3 | 1.7 | | |
| Q33 During your current or most recent period NOT experiencing a flare, how often do/did you have to cancel or reschedule an engagement, meeting, etc. because of your bowel disease? | | | | |
| Always (100% of the time) | 1 | | 0.6 | |
| Most of the time （75%–99%） | 3 | | 1.7 | |
| Much of the time (50%–74%) | 6 | | 3.5 | |
| Sometimes (25%–49%) | 26 | | 15.1 | |
| Hardly ever/never (less than 25% of the time) | 102 | | 59.3 | |
| Not applicable/don’t know | 30 | | 17.4 | |
| No answer | 4 | | 2.3 | |
| Q34 During your current or most recent period NOT experiencing a flare, how often do/did you worry about when the next flare will strike? | | | | |
| Always (100% of the time) | 35 | 20.3 | | |
| Most of the time （75%–99%） | 19 | 11.0 | | |
| Much of the time (50%–74%) | 19 | 11.0 | | |
| Sometimes (25%–49%) | 53 | 30.8 | | |
| Hardly ever/never (less than 25% of the time) | 28 | 16.3 | | |
| Not applicable/don’t know | 13 | 7.6 | | |
| No answer | 5 | 2.9 | | |
| Q35 Do you feel stressed or pressured about sick time from work due to IBD? | | | | |
| Yes | 124 | | 72.1 | |
| No | 18 | | 10.5 | |
| Not applicable/don’t know | 25 | | 14.5 | |
| No answer | 5 | | 2.9 | |
| Q37 In the past year, how many days have you been absent from work for reasons related to your IBD? | | | | |
| 0 day | 47 | | | 27.3 |
| 1 day | 9 | | | 5.2 |
| 2 days | 9 | | | 5.2 |
| 3 days | 8 | | | 4.7 |
| 4 days | 1 | | | 0.6 |
| 5 days | 10 | | | 5.8 |
| 6-10 days | 20 | | | 11.6 |
| 11-15 days | 19 | | | 11.0 |
| 16-20 days | 7 | | | 4.1 |
| 20-25 days | 3 | | | 1.7 |
| More than 25 days | 25 | | | 14.5 |
| No answer | 14 | | | 8.1 |

IBD, inflammatory bowel disease.

**Table S3. The impact of IBD on intimate relationships (N=172)**

|  | No. | % |
| --- | --- | --- |
| Q44 My IBD has prevented me from pursuing intimate relationship(s) | | |
| Strongly agree | 15 | 8.7 |
| Agree | 29 | 16.9 |
| Neither agree nor disagree | 49 | 28.5 |
| Disagree | 24 | 14.0 |
| Strongly disagree | 23 | 13.4 |
| Not applicable | 26 | 15.1 |
| No answer | 6 | 3.5 |
| Q45 My IBD has caused an intimate relationship to end | | |
| Strongly agree | 14 | 8.1 |
| Agree | 24 | 14.0 |
| Neither agree nor disagree | 42 | 24.4 |
| Disagree | 25 | 14.5 |
| Strongly disagree | 20 | 11.6 |
| Not applicable | 40 | 23.3 |
| No answer | 7 | 4.1 |
| Q46 My IBD has prevented me from making new and/or keeping friends | | |
| Strongly agree | 12 | 7.0 |
| Agree | 28 | 16.3 |
| Neither agree nor disagree | 40 | 23.3 |
| Disagree | 27 | 15.7 |
| Strongly disagree | 29 | 16.9 |
| Not applicable | 29 | 16.9 |
| No answer | 7 | 4.1 |
| Q52 To what degree has being part of a patient's association affected the impact on your life in general as someone with IBD? | | |
| Significant improvement | 30 | 17.4 |
| Slight improvement | 57 | 33.1 |
| No change | 30 | 17.4 |
| Slightly worse | 3 | 1.7 |
| Not applicable/don’t know | 14 | 8.1 |
| No answer | 38 | 22.1 |

IBD, inflammatory bowel disease.

**Table S4. Overall life impact (N=172)**

|  | No. | | | % | | |
| --- | --- | --- | --- | --- | --- | --- |
| Q48 Please tick any of the following that apply to you: | | | | | | |
|  | Applicable No. (%) | | Not applicable No. (%) | | | No answer No. (%) |
| I frequently consider the availability of toilets whenever I plan to attend an event, meeting, etc. | 108 (62.8) | | 44 (25.6) | | | 20 (11.6) |
| I worry about the ready availability of a toilet whenever I go somewhere new | 124 (72.1) | | 28 (16.3) | | | 20 (11.6) |
| I keep a list (from memory or written) of clean, accessible toilets and consider this when I leave home | 30 (17.4) | | 122 (70.9) | | | 20 (11.6) |
| I have had to be rude to people at times in order gain access to a toilet in order to  prevent an accident | 58 (33.7) | | 94 (54.7) | | | 20 (11.6) |
| Other people have sometimes joked about me urgently needing a toilet | 10 (5.8) | | 142 (82.6) | | | 20 (11.6) |
| I frequently wake from sleeping as a result of pain from my IBD | 43 (25) | | 109 (63.4) | | | 20 (11.6) |
| Q49 The first time in your life when you met another person with IBD, did you find that this meeting made you feel: | | | | | | |
| More optimistic | | 61 | | | 35.5 | |
| More pessimistic | | 14 | | | 8.1 | |
| Neither more optimistic nor more pessimistic | | 88 | | | 51.2 | |
| No answer | | 9 | | | 5.2 | |
| Q50 Have you engaged in any way with one of IBD patient associations? | | | | | | |
| No | | 39 | | | 22.7 | |
| Yes | | 130 | | | 75.6 | |
| No answer | | 3 | | | 1.7 | |
| Q51 Have you engaged in any way with one of IBD patient associations? If so, what level of involvement do you have? (Tick all that apply) | | | | | | |
|  | Applicable No. (%) | | Not applicable No. (%) | | | No answer No. (%) |
| Attending local or national patient meetings | 73 (42.4) | | 90 (52.3) | | | 9(5.2) |
| Signing-up to be a member of your national IBD association | 108 (62.8) | | 55 (32.0) | | | 9(5.2) |
| Receiving patient information leaflets from your national IBD association | 73 (42.4) | | 90 (52.3) | | | 9(5.2) |
| Calling a helpline, or emailing your national IBD association | 13 (7.6) | | 150 (87.2) | | | 9(5.2) |
| Subscribing to newsletters or magazines from your national IBD association | 58 (33.7) | | 105 (61.0) | | | 9(5.2) |
| Volunteering to help your national IBD association | 39 (22.7) | | 124 (72.1) | | | 9(5.2) |
| Helping your national IBD association in fundraising | 13 (7.6) | | 150 (87.2) | | | 9(5.2) |
| Becoming a leader, or joining a committee within your national IBD association | 38 (22.1) | | 125 (72.7) | | | 9(5.2) |
| Becoming an EFCCA delegate, or working within an EFCCA project team | 2 (1.2) | | 161 (93.6) | | | 9(5.2) |

IBD, inflammatory bowel disease.
